# The prevalence of hepatitis B in Chinese general population from 2018 to 2022: a systematic review and meta-analysis

**DOI:** 10.1186/s12879-024-09103-8

**Published:** 2024-02-16

**Authors:** Shuwen Bai, Wen Dang, Wenying Hong, Wenyu Liao, Robert David Smith

**Affiliations:** Department of Public Health and Medicinal Administration, Faculty of Health Sciences, University of Macau, Macao SAR, China

**Keywords:** Hepatitis B, HBsAg, Prevalence, Infection, China, Meta-analysis

## Abstract

**Background:**

Within China, Hepatitis B virus (HBV) infection remains widely prevalent and one of the major public health problems. There have been only two previous estimates of its prevalence at the population level in China, with the latest survey conducted in 2006. A meta-analysis estimated the prevalence of HBV within China between 2013 and 2017 as 7%. This review provides an updated estimate of HBV prevalence in China from 2018 to 2022.

**Methods:**

Systematic searches of literature from January 1, 2018 to December 25, 2022 were conducted in four international databases (Medline, Web of Science, Embase, Cochrane Database of Systematic Reviews) and three Chinese databases (CNKI, CBM, and WanFang data). Random-effects meta-analyses were conducted to calculate the pooled HBV prevalence with 95% confidence intervals in the overall population and subgroups. Publication bias, heterogeneity between studies, and study quality were assessed.

**Results:**

Twenty-five articles were included in the meta-analysis. The pooled prevalence of HBV infection in the Chinese general population from 2018 to 2022 was 3% (95%CI: 2–4%). The prevalence of HBV infection was similar between males and females (both 3%), while rural areas had a higher prevalence than urban areas (3% vs 2%). The highest prevalence of HBV was reported in the eastern provinces (4, 95%CI: 2–6%). The HBV prevalence of people aged ≥18 years old (6, 95%CI: 4–8%) was higher than people aged < 18 years old (0, 95%CI: 0–1%).

**Conclusion:**

Compared to the previous meta-analysis prevalence in 2013–2017, the updated meta-analysis estimated prevalence of HBV infection (3%) from 2018 to 2020 showed a decreasing trend, suggesting China had moved into a lower intermediate epidemic area (2–5%). However, the prevalence of HBV in rural areas and eastern regions was still higher than the national average. People aged ≥18 years old showed a higher HBV prevalence. HBV prevention should be prioritized in the highest-prevalence areas and high-risk populations. Due to heterogeneity in data collection methods among studies, there remains a need for systematic surveillance of nationwide HBV prevalence.

**Supplementary Information:**

The online version contains supplementary material available at 10.1186/s12879-024-09103-8.

## Background

Hepatitis B virus (HBV) is a leading cause of liver cancer, with an estimated 296 million people living with chronic HBV infection worldwide in 2019 and 1.5 million new infections occurring each year [1]. Due to the increased risks of morbidity after infection, HBV was predicted to be the tenth leading cause of death globally, accounting for 820,000 deaths in 2019 [2]. HBV infection is most prevalent in Africa, with a prevalence rate of 8.83%, followed by the Western Pacific at 5.26% [3]. Definitions of global prevalence rates for HBV are separated into four categories: high (> 8%), intermediate (5–7.99%), medium-low (2–4.99%), and low HBV endemic (< 2%) prevalence [4–6].

China has been reported to have a relatively high prevalence of HBV, although during the past 30 years, the prevalence of HBV infection showed a decreasing trend in China. Two population-level surveys of HBV infection rates in Chinese people aged 1–59 showed that the prevalence of HBV decreased between 1992 and 2006 from 9.75% in 1992 to 7.18%. Additionally, a downward trend was identified among people aged 1–29, from 10.1% in 1992 to 2.6% in 2014 [7]. Over this period, the HBV immunization programs were credited with the observed reductions in HBV prevalence [8]. Estimates on the HBV prevalence in China remains unclear, as previous meta-analyses have found high heterogeneity in reported prevalence across studies [9]. An explanation for the high variability in prevalence among studies could be different in population types. For example, in one survey, the prevalence of HBV among Chinese pregnant women was estimated to be 6.17% [10], compared to another survey was 3.3% [11]. Within China, there are still sub-groups exhibiting a higher risk of HBV infection. A previous meta-analysis estimated the HBV prevalence in China as 6.89% during the period between 2013 and 2017 [9]. Approximately 90% of those infected HBV cases were adults aged 20 years and over. The prevalence rate was higher in males compared to females, higher in rural areas than in urban areas, and higher in western regions than in central and eastern regions. The same meta-analysis investigated geographical differences within China and estimated the prevalence rates in western China were 8.9%, followed by eastern China (6.2%) and central China (5.2%) [9]. The last synthesis of data describing nationwide HBV prevalence was published in 2017. We performed an updated systematic review and meta-analysis to identify new studies published since 2018 and to describe trends in HBV prevalence in China.

## Methods

### Search strategy

This article was conducted according to the Preferred Reporting Items for Systematic Reviews and Meta-Analyses (PRISMA) statement guidelines [12]. The protocol of this systematic review was registered in the International prospective register of systematic reviews (PROSPERO), National Institute for Health research (Number: CRD42023396671). This review provided an update on the findings of a previous systematic review [9]. Only articles published from January 1, 2018 to December 25, 2022 were retrieved*.* Databases searched included four international databases (Medline, Web of Science, Cochrane Database of Systematic Reviews, and Embase) and three national databases (China National Knowledge Infrastructure (CNKI), Chinese Biomedical Database (CBM), and WanFang Data). All included search terms used for each searched database were reported in the Additional file 1.

### Inclusion and exclusion criteria

All articles that reported cross-sectional studies on the general population tested for HBsAg in different regions of China were included. As this was an updated systematic review from a previous systematic review covering up to 2017 [9], only articles published and covering data from 2018 and 2022 were included. The inclusion criteria were restricted to original research articles written in English or Chinese. Studies were limited to those recruited using random sampling approaches to minimize the risk of section bias in the estimates of HBV prevalence rates. We only retained studies with sample sizes above 800 participants, as this was estimated by the review team to be a reasonable sample size for population-level studies and to minimize bias from smaller studies on the pooled HBV prevalence. To ensure the validity of our meta-analysis findings, the surveillance reports were excluded due to insufficient reporting of data sources and variations in data collection methods or statistical approaches across different reports. In this article, the proportion of participants with a positive HBsAg positive result was determined as the criteria for HBV cases. The exclusion criteria were the following: (1) conference abstracts, case reports, surveillance reports, and systematic reviews or meta-analyses; (2) study designs with non-random sampling; (3) studies that did not report the positive rate of HBsAg; (4) sample sizes of less than 800 people; (5) study populations co-infected with, Hepatitis C Virus (HCV) or Human Immunodeficiency Virus (HIV). While we excluded review articles, the citations of identified systematic reviews or meta-analyses were examined for additional eligible studies.

### Screening, data extraction and quality assessment

Articles were screened independently by the primary reviewer and at least one secondary reviewer, with all conflicting decisions agreed up between reviewers. After the title and abstract were screened, the full text was screened, with the study characteristics of the included article extracted. Study characteristics included author, publication year, the number of HBV positive cases, sample size, geographical regions surveyed (provinces, autonomous regions, or municipalities), urban/rural status, sex, age groups, HBV testing method (e.g. Enzyme-Linked Immunosorbent Assay) of the included papers.

The Cross-Sectional/Prevalence Study Quality Assessment Forms which were recommended by the Agency for Healthcare Research and Quality (AHRQ) [13] were used to assess study methodological quality. The AHRQ (Additional file 2: Table S1), consisted of 11 items, of which 10 questions related to cross-sectional studies. The 11th question designated for longitudinal data studies was not applicable in this review, so it was excluded. Items 1 to 10 were graded was scored using 3 options: Yes, No, and Unclear. If the answer was Yes, the item got 1 point, and if the answer was No or Unclear, the item got 0 points. The AHRQ was scored using a sum score of the first 10 items, with higher scores indicating higher levels of methodological quality.

### Statistical analysis

All statistical analyses were conducted using R software version 4.2.3 [14]. The primary analysis estimated the pooled prevalence of HBsAg positive cases, reported as a percentage with the 95% confidence interval (95% CI) using a random-effects meta-analysis. In subgroups analyses, pooled prevalence rates were assessed within prespecified subgroups by sexes, different regions, urban/rural status, and age groups. Subgroup analyses were conducted in different regions. According to the National Bureau of Statistics [15], Mainland China was divided into four major regions based on economic development zones, namely the eastern, central, western, and northeast regions. For subgroup analysis of sexes were separated into males and females. For subgroup analysis of urban/rural status, we described this according to the rural/urban distinction of the original included literature. Rural and urban areas in the original included literature were divided according to the National Bureau of Statistics of China [16]. For subgroup analysis of age groups, we divided the age groups into two parts which are people aged < 18 years old and aged ≥18 years old. The division of the age groups mainly based on a nationwide HBV vaccination program 18 years ago in China in 2005, providing free HBV vaccine to all newborns [17]. This division allowed us to intuitively analyze the comparison between different age groups.

Magnitude of heterogeneity was estimated by *I*^2^. *I*^2^ > 50% indicated high heterogeneity. Sources of heterogeneity were investigated within prespecified subgroup analysis of different regions, urban/rural status, sexes, and age groups, which were extracted from the included articles. Publication bias assessment was tested by Egger’s test and funnel plot. The Freeman-Tukey double arcsine transformation was applied in funnel plot analysis to stabilize variance and improve result accuracy [18]. Egger’s test, through linear regression analysis of the funnel plot, determined the presence of publication bias, with a *P* value greater than 0.05 indicating no significant bias [19].

## Results

**Fig. 1:**
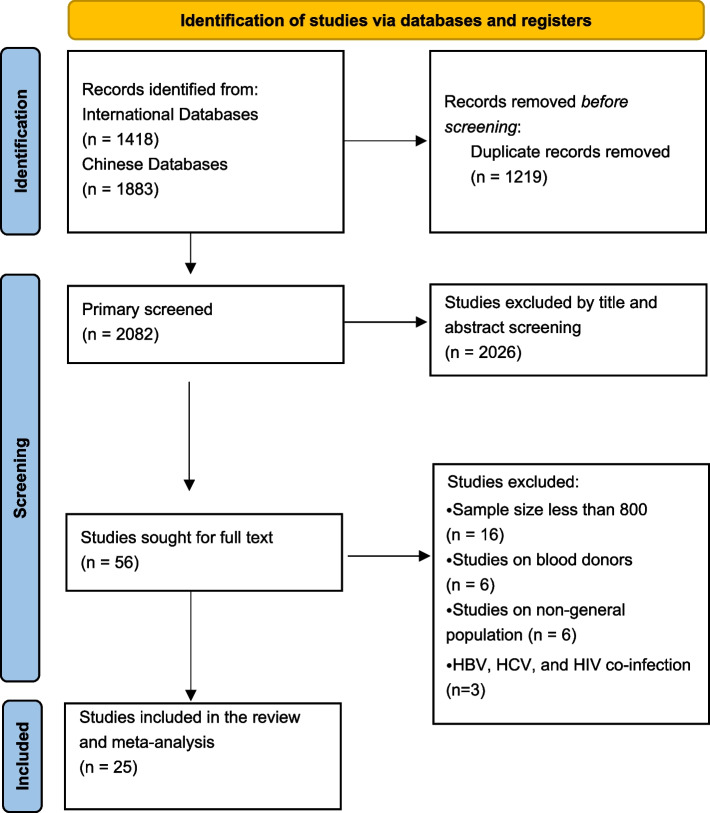
PRISMA flow chart. Footnote: HBV, Hepatitis B virus; HCV, Hepatitis C Virus; HIV, Human Immunodeficiency Virus; PRISMA, Preferred Reporting Items for Systematic Reviews and Meta-Analyses

Across the seven searched databases, a total of 1418 articles published in English and 1883 articles published in Chinese were retrieved. After removing duplicate articles (691 Chinese and 528 English respectively), there were 2082 articles remaining. Of retrieved articles, 2026 (61%) articles were removed after title and abstract screening, and further 31 articles were removed during the full text screening. Finally, a total of 25 (1%) articles were included in this review (Fig.1). The characteristics of all the included articles [20–44] were shown in Table 1 and the characteristics of age groups were shown in the Additional file 3.

**Table 1:** Characteristics of the studies included in the meta-analysis

First author	Publication Year	Geographical regions	Male HBsAg positive/Male	Female HBsAg positive/Female	Rural HBsAg positive/Rural status	Urban HBsAg positive/Urban status	Sample Size(N)	HBsAg Positive (n)	HBV Testing Method	Quality Grade
He Z [20]	2019	Yun Nan Province	NA/1523	NA/2099	NA/NA	NA/NA	3622	41	ELISA	5
Sun CW [21]	2019	A city district	7/1304	12/1064	16/1456	3/912	2368	19	CMIA	5
He Z [22]	2020	Yun Nan Province	NA/NA	45/2063	45/2063	NA/NA	2063	45	ELISA	5
Deng QY [23]	2020	Guang Xi Province	200/4567	238/5689	NA/NA	NA/NA	10,256	438	CMIA	4
Li GD [24]	2020	An Hui Province	NA/921	NA/836	NA/NA	NA/NA	1757	124	ELISA	5
Shi W [25]	2020	Zhe Jiang Province	90/1941	102/2253	NA/NA	NA/NA	4194	192	ELISA	5
Li JR [26]	2021	Yun Nan Province	NA/NA	NA/NA	NA/NA	NA/NA	5448	35	ELISA	5
Li JR [27]	2021	Yun Nan Province	NA/NA	NA/NA	NA/NA	NA/NA	21,177	79	ELISA	4
Wang Q [28]	2021	Chong Qing City	NA/NA	NA/NA	31/387	15/449	836	46	ELISA	5
Xu NN [29]	2021	Zhe Jiang Province	8/2014	7/1803	NA/NA	NA/NA	3817	15	CMIA	4
Yang QY [30]	2021	Guang Dong Province	118/1904	116/2076	NA/NA	NA/NA	3980	234	CLEIA	5
Yang XH [31]	2021	Fu Jian Province	220/3662	299/4736	NA/NA	NA/NA	8398	519	ELISA	4
Zhang SY [32]	2021	He Nan Province	2/659	8/991	6/990	4/660	1650	10	ELISA	4
M. Jiang [33]	2021	Zhe Jiang Province	NA/NA	NA/NA	NA/NA	NA/NA	1849	25	CLEIA	5
Ci P [34]	2022	Tibet Province	23/543	16/530	NA/NA	NA/NA	1073	39	ELISA	5
Deng XY [35]	2022	Jiang Su Province	NA/NA	NA/NA	NA/NA	NA/NA	4502	130	ELISA+CMIA	5
Guo ZZ [36]	2022	Qing Hai Province	8/756	12/905	NA/NA	NA/NA	1661	20	ELISA	4
Huang QM [37]	2022	Jiang Su Province	10/441	25/569	NA/NA	NA/NA	1010	35	ELISA	4
Li WQ [38]	2022	He Nan Province	3/981	11/1329	NA/NA	NA/NA	2310	14	ELISA	6
Liang Y [39]	2022	He Nan Province	NA/NA	NA/NA	NA/NA	NA/NA	1854	13	ELISA	5
Liu LJ [40]	2022	Si Chuang Province	4/484	12/484	11/585	5/383	968	16	CMIA	5
Wu FY [41]	2022	Jiang Xi Province	190/2045	222/3074	234/2507	178/2612	5119	412	ELISA	4
Xie CZ [42]	2022	Shan Dong Province	164/2110	109/1945	NA/NA	NA/NA	4055	273	ELISA	4
Yang JX [43]	2022	Guang Xi Province	31/450	46/811	NA/NA	NA/NA	1261	77	ELISA	4
Zhang T [44]	2022	Hu Bei Province	70/1872	95/2716	NA/NA	NA/NA	4588	165	ELISA	5

*NA* stands for ‘not applicable’ due to the absence of data

*ELISA* stands for Enzyme-Linked Immunosorbent Assay

*CMIA* stands for CLIA-Chemiluminescence Immunoassay

All included studies accounted for a total sample size of 99,816 participants with a total number of HBsAg positive cases of 3016 (3%). The largest study sample size included in this review included 21,177 participants and the smallest included 836 participants. In terms of quality assessment, all the studies (100%) had a total quality of more than 4 points, 14 (56%) studies had a total quality of 5 points, and one article (4%) had a total quality of more than 5 points (Additional file 4: Table S3).

### Prevalence of HBV infection in the general population

**Fig. 2:**
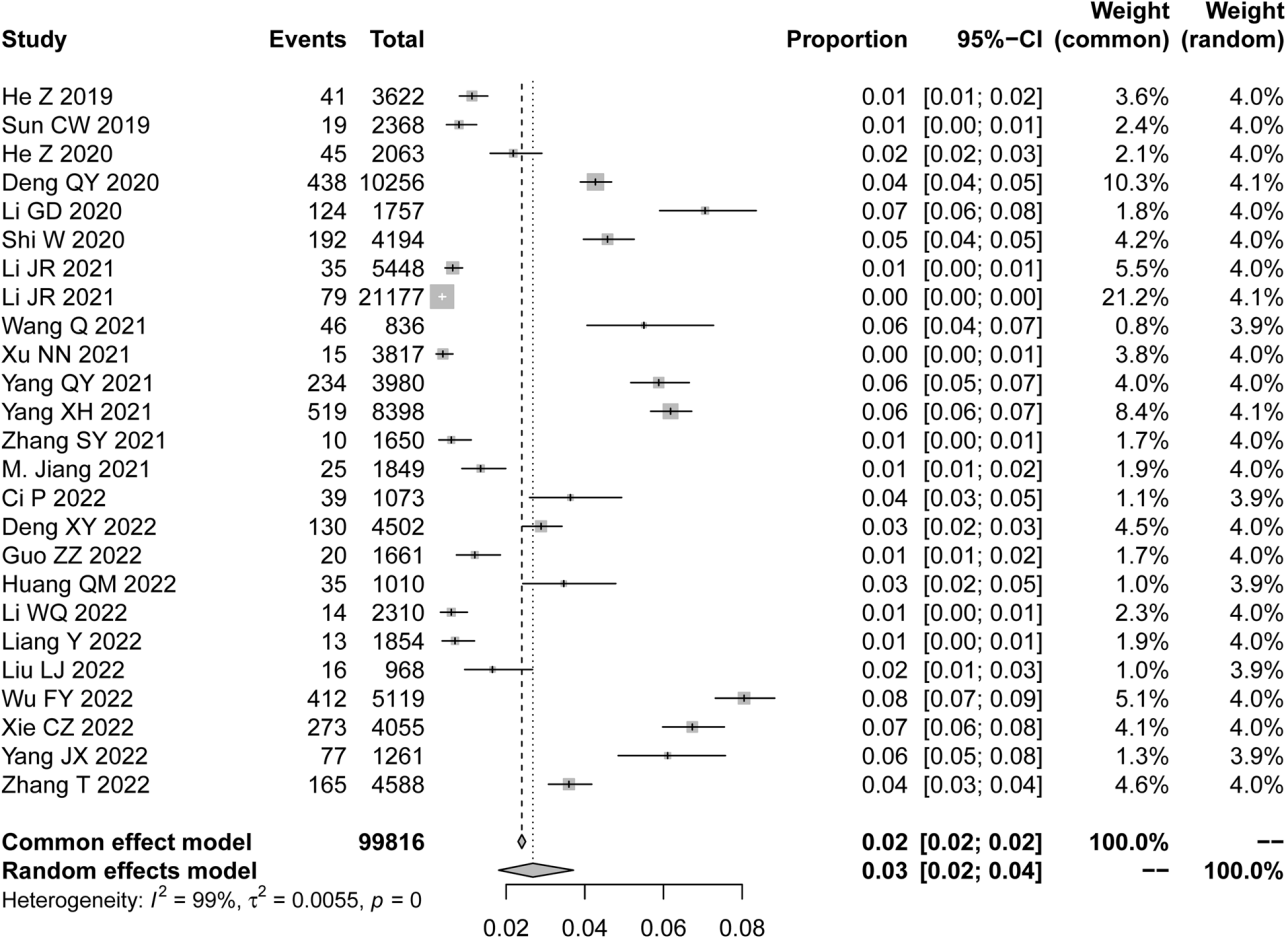
Forest plot of HBV prevalence in the general population from 2018 to 2022

The prevalence of HBsAg positive in China from 2018 to 2022 was 3% (95%CI, 2–4%) (Fig. 2). The highest prevalence was reported in Jiangxi Province (Central China), with a prevalence rate of 8% (95%CI, 7–9%) [41].

**Fig. 3:**
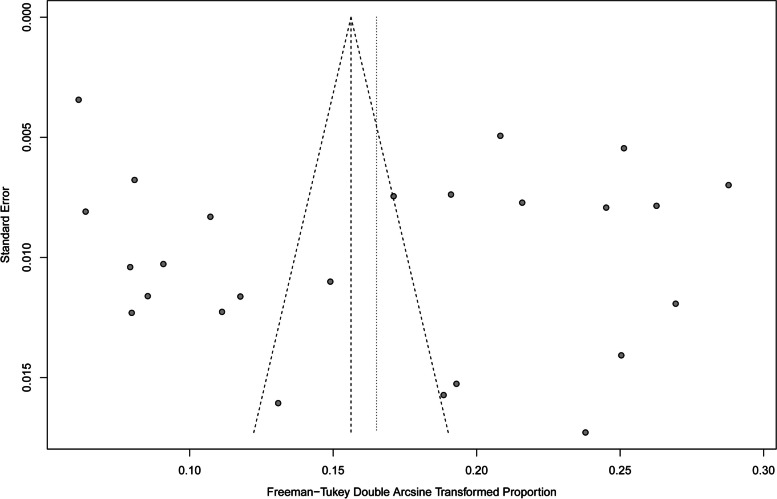
Bias assessment funnel plot of studies reporting HBV prevalence in China from 2018 to 2022

**Fig. 4:**
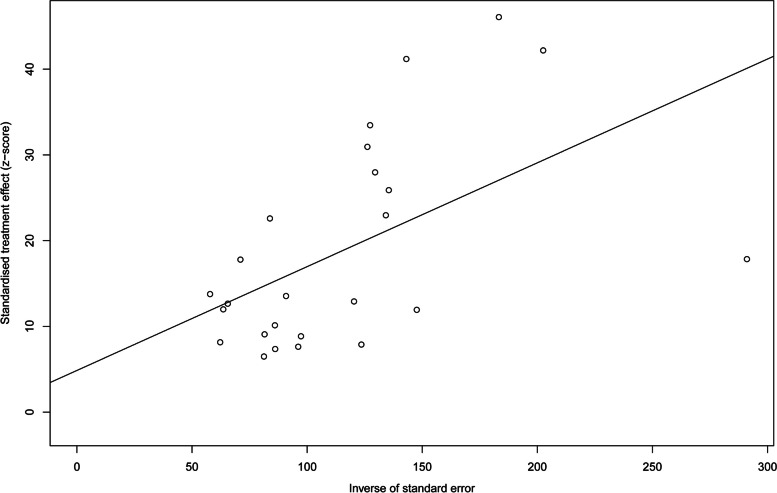
Egger’s test

Heterogeneity of the meta-analysis was substantial above the *I*^2^ = 50% cutoff (*I*^2^ = 99%, *P* < 0.01). Based on the funnel plot displaying a moderately symmetrical distribution of studies across the funnel (Fig. 3) and Egger’s test (Fig. 4) for the prevalence of HBV infection (*P* = 0.35), there was little evidence of possible publication bias among all studies.

### Subgroup analysis of HBV infection according to sexes, regions, urban/rural status, and age groups

Table 2 displays the results of the subgroup analysis. In the subgroup analysis of sexes, HBsAg positive cases in males were reported in 16 articles and females was reported in 17 articles. The prevalence of female (3, 95%CI: 2, 4%) was similar to male (3, 95%CI: 2, 5%). The heterogeneity within both analyses of sexes remained substantial (*I*^2^ = 97 and 98%, respectively).

**Table 2:** Sub-group meta-analysis of studies from 2018 to 2022

Sub-groups	Numbers of Study	Prevalence (%)	95%CI	*I*^2^(%)	*P*-value	Egger’s test *P*-value
Region						
Eastern	8	4	(2, 6%)	99	*P* < 0.01	–
Middle	6	3	(1, 6%)	99	*P* < 0.01	–
Western	10	2	(1, 4%)	99	*P* < 0.01	0.17
Sex						
Male	16	3	(2, 5%)	98	*P* < 0.01	0.12
Female	17	3	(2, 4%)	97	*P* < 0.01	0.06
Urban/Rural						
Urban	5	2	(0, 5%)	97	*P* < 0.01	–
Rural	6	3	(1, 6%)	98	*P* < 0.01	–
Age groups						
≥18 years old	14	6	(4, 8%)	98	*P* < 0.01	0.04
< 18 years old	17	0	(0, 1%)	89	*P* < 0.01	0.48

**Fig. 5:**
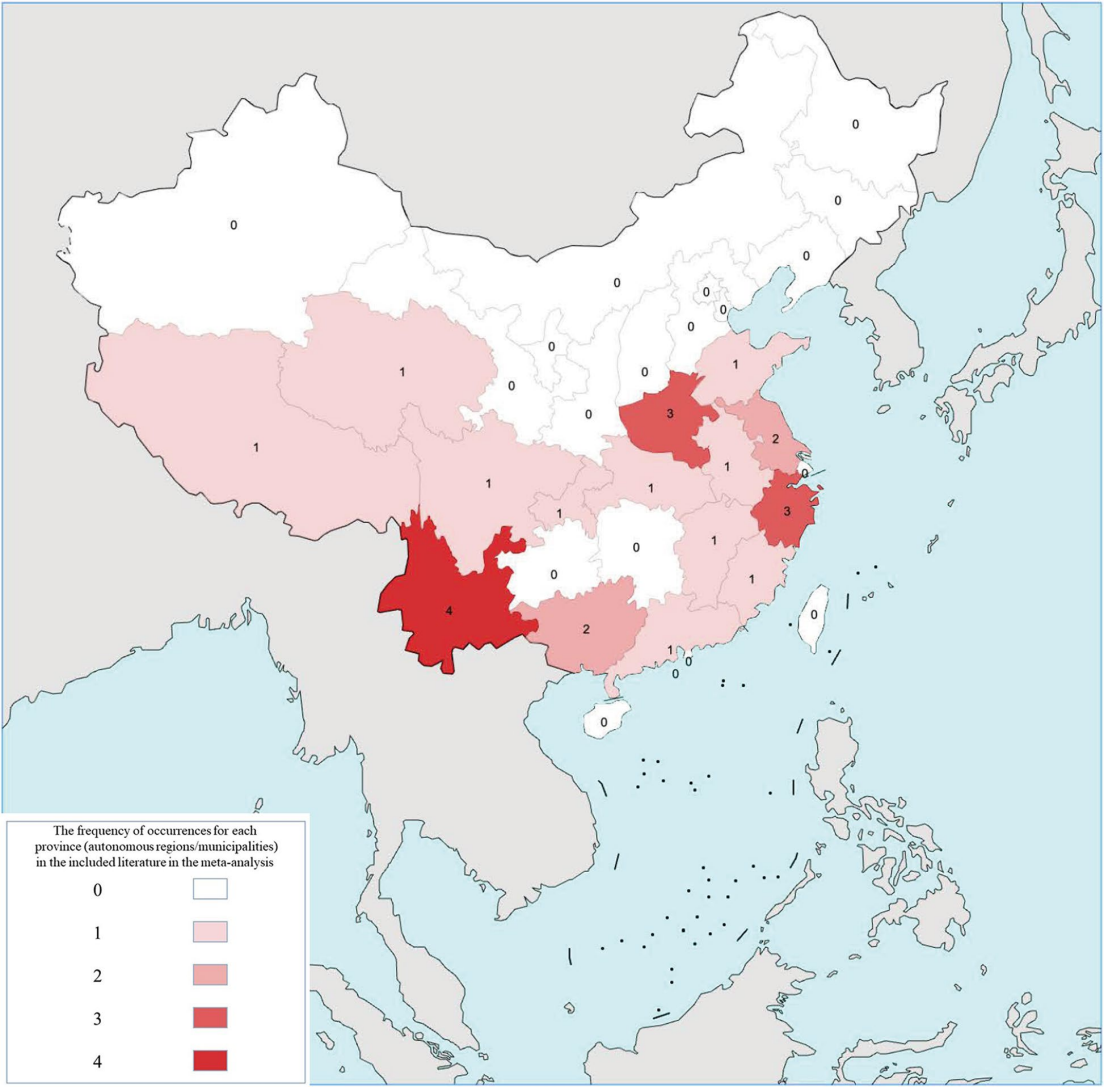
The frequency of occurrences for each province, autonomous regions, and municipalities in the included literature in the meta-analysis

In the regional subgroup, HBsAg positive cases in eastern provinces were reported in 8 articles, western provinces were reported in 10 articles, and central provinces were reported in 6 articles. The eastern region (4, 95%CI: 2–6%) had the highest prevalence, followed by the central region (3, 95%CI:1–6%) and the western region (2, 95%CI: 1–4%). Heterogeneity within the analysis of the eastern, central, and western regions remained substantial (*I*^2^ = 99% in each region). None of the included articles in this meta-analysis reported on HBV prevalence within the northeast region (as shown in Fig. 5).

As for the subgroup of urban/rural status, HBsAg positive cases in urban status were reported in 5 articles and rural status were reported in 6 articles. The prevalence in urban (2, 95%CI: 0–5%) was lower than that in rural (3, 95%CI: 1–6%). The heterogeneity was substantial at *I*^2^ = 97 and 98%, respectively.

As for the subgroup of age groups, HBsAg positive cases in people aged ≥18 years old were reported in 14 articles and people < 18 years old were reported in 17 articles. The prevalence of people aged ≥18 years old (6, 95%CI: 4–8%) was higher than people aged less < 18 years old (0, 95%CI: 0–1%). Heterogeneity in both age groups remained substantial at *I*^2^ = 98 and 89%.

## Discussion

We systematically evaluated peer-reviewed studies published in English or Chinese describing HBV prevalence in China from 2018 to 2022 to provide an updated meta-analysis from a previous meta-analysis. Using data from 25 included studies, our meta-analysis found the pooled prevalence of HBV was 3%, suggesting that China now has gone from a higher-intermediate to a lower-intermediate prevalence country over the past decade [5, 6]. In the subgroup analysis, we found that HBV prevalence was similar between males and females. The prevalence of HBV appeared to be higher in the eastern regions compared to other regions and higher in rural areas compared to urban areas. After comparison between the people aged < 18 years old and those aged ≥18 years old, people aged ≥18 years old had the higher HBV prevalence.

The previous 2017 meta-analysis describing HBV prevalence in China included a total sample size of 5,422,405 individuals across 27 studies, including four large nationwide studies with participants from all 31 provinces [9]. In contrast, our meta-analysis included 99,816 individuals across 25 studies, all of which were local or regional studies and none were conducted at the national level. The absence of nationwide studies in our meta-analysis may limit the generalizability of our findings to the entire country. In the earlier review, 16 studies included rural HBV prevalence and 10 reported urban prevalence [9]. In this presented review, there were 6 studies on rural prevalence and 5 studies on urban prevalence. This presented review had fewer studies on the prevalence of HBV in rural and urban areas, which would lead to a reduction in HBV prevalence in rural and urban areas. However, we found a comparable amount of studies reporting on both rural and urban areas, which enhanced the comparability of our findings for rural and urban areas in our meta-analysis. The previous review (16 studies) [9] had more studies conducted within the eastern region of China compared to the current review (8 studies). This may potentially restrict the generalizability of our findings regarding HBV prevalence in the eastern region. The previous 2017 meta-analysis divided the age groups into 9 groups but in our meta-analysis, we only had two age groups. However, we both found the prevalence of HBV increased with age.

Our meta-analysis and previous meta-analysis identified similar prevalence of HBV in both males and females. Compared to the previous meta-analysis, it appears the prevalence rates of HBV in both men and women have decreased. Since 2012, universal screening for HBsAg in pregnant women has been implemented per national policy in China, leading to improved detection and treatment rates for women [45]. Previously, it was shown that males had a higher risk of contracting HBV [46]. Other investigations on sex and risk of HBV attributed to males relatively greater exposure to sexually transmitted risk factors, such as men who have sex with men and persons with multiple sexual partners to increased prevalence of HBV compared to females [47, 48]. However, in recent years, this higher prevalence compared to females appears to have decreased, likely due to increased awareness and access to preventative healthcare [49, 50].

In our subgroup analysis, the prevalence of HBV was highest among studies in the eastern regions of China. However, the 2013–2017 data showed that the western region had the highest prevalence, while both studies indicated the central region had the lowest prevalence. There are several possible reasons why our analysis and the 2017 review identified different patterns in HBV prevalence by region. In recent years, large-scale population migration has occurred, with Chinese eastern coastal areas seeing increasing rates of migration to those regions [51]. According to the ‘National Survey Report on Migrant Workers in 2018’, there was a net inflow of 53.98 million rural migrant workers into the eastern region [51]. As a result of the large-scale migration, a substantial number of HBV carriers, infected individuals, and those without HBV antibodies could have migrated to those areas. A survey collecting data on the prevalence of HBV among rural males aged 21–49 in China from 2010 to 2012 revealed an HBsAg positive prevalence of 6% and a 26% prevalence rate of HBeAg (indicative of high susceptibility of infection) [52]. Inequalities in access to medical care in different regions may lead to poorer medical testing levels in underdeveloped areas, making HBV surveys challenging [53].

Both this meta-analysis and the previous meta-analysis reported a higher prevalence in rural areas compared to urban areas. Reduced health literacy about HBV, poorer access to HBV treatment and disparities in HBV vaccination coverage among rural residents may account for this observed increased prevalence. In 2015, the World Health Organization recommended that the management of HBV infection should involve connecting individuals to care through the process of detection and diagnosis [54]. However, in a survey of chronic hepatitis B (CHB) among rural Chinese women, nine out of 10 women with CHB were unaware of their infection status, within this survey 7.4% required antiviral treatment, only 0.22% received treatment, and 29% of women with CHB also tested positive for HBeAg (indicating high infectivity) [55, 56]. Compared to urban population, there is generally a lack of widespread health education and awareness regarding HBV, leading to a higher prevalence of HBV among rural residents [57]. Furthermore, since HBV vaccines in China are not provided free of charge for adults, urban residents are typically more capable of affording vaccination compared to the rural people [58, 59]. To address this, the Chinese government began providing free vaccination to newborns and children since 2005, resulting in a significant increase in vaccine coverage rates [60]. This increase was identified in both urban areas, where coverage reached 95%, and rural areas, where coverage rates climbed to between 84 and 97% [61].

The prevalence of HBV varied in different age groups. In our meta-analysis, the age group with higher prevalence was people aged ≥18 years old, while the age group with lower prevalence was those aged < 18 years old (6 and < 1% respectively). This result indicated that the prevalence of HBV may increase with age, which was concurrent with the results in the 2017 meta-analysis [9]. Possible reasons for this result include changes in vaccination policies for younger age groups and improvements in screening practice. The HBV vaccine has been available in China since 1982 [17], but there has been limited availability [62], with varying vaccination rates among different regions of China [60], partially due to vaccine pricing [63]. These reasons led to the vaccination rate of HBV in regions of China being limited since the vaccine was introduced. The Chinese government has implemented a nationwide HBV program, introduced in 2005, that provides vaccines free of charge to all neonates, leading to a rapid increase in HBV immunization coverage among young children [17]. Therefore, the changes in HBV vaccine policies over different years could account for the differences in prevalence that were observed in aging. Detection through screening has also increased over recent years, nucleic acid amplification testing (NAT) was included in routine donor screenings in 2010 [64] and this practice was expanded nationwide in 2014 [65]. Full coverage of NAT in all blood stations substantially improved blood safety and prevented transfusion-transmitted infections, including HBV [66].

Over the past 30 years, numerous strategies addressing HBV have been implemented within China and the prevalence of HBV has been declining during this period. The Chinese government allocated funds for newborn vaccination and procured national immunization vaccines and syringes between 1992 and 2005 [8]. A catch-up vaccination campaign for children under 15 years old vaccinated approximately 68 million children was conducted between 2009 and 2011 [67]. Pregnant women and patients undergoing medical procedures were required to be screened for HBV. A pre-pregnancy health screening program was launched in 2010, offering free HBV serological testing to couples of childbearing ages, and expanded nationwide in 2013 [68]. As a result, HBV three-dose vaccine and timely birth-dose coverage in China have achieved the targeted service coverage of 90% for elimination of HBV in 2015 [68]. China has made significant strides in reducing HBV prevalence, but internationally, the prevalence remains relatively high compared to other countries. In England [69] and Canada [53], the general population’s prevalence of HBV is low at 0.5–1.0%, and in Northern Europe, it is less than 0.1% [53]. Both current and previous analyses noted high prevalence in rural areas, which could be due to unequal distribution of medical resources, varying diagnostic capabilities, and lack of free adult HBV vaccination [53]. Further researchers can focus on reducing vaccination for hard-to-reach populations through improving medical service coverage and reducing health inequalities. Wider implementation of a more comprehensive HBV vaccination program for adults, especially those living in less economically developed areas, could also address the higher infection prevalence among older age groups. It is also important to improve diagnostic capacity and provide timely treatment for HBV patients in order to reduce the prevalence of HBV.

This systematic review had some limitations. First, the heterogeneity of meta-analysis was high. Subgroup analysis was used to identify potential sources of heterogeneity and provided data on HBV infection rates among sub-groups. However, heterogeneity in the subgroup analysis did not decrease substantially. Additionally, due to the lack of national-scale surveys, the results of the meta-analyses may not reflect the true prevalence of HBV in the general population in China. Moreover, only cross-sectional studies that reported HBV prevalence were analyzed in this meta-analysis. Therefore, this analysis does not report HBV incidence and cannot evaluate which public health programs may have contributed to observed decreases in HBV prevalence. Further research may consider other factors that may explain the source of high heterogeneity among studies on HBV prevalence in China. One way to address this is to use individual-level data from a large population across China. The provincial and city scale surveys of HBV prevalence in the general population in China could be expanded, where possible. Only the prevalence of HBV in the general population was studied in this study, and the prevalence of HBV in people within special disease groups or high/low-risk groups may be investigated in further studies.

## Conclusion

This meta-analysis of currently available evidence from 2018 to 2022 indicates that China has experienced a decline in HBV prevalence, transitioning from a higher intermediate area to a medium-low epidemic level. However, in the subgroup analysis by different regions and age groups, the prevalence of HBV showed significant differences, especially among populations in rural areas and people aged ≥18 years old, where the prevalence of HBV was higher. This suggests that in the future, HBV prevention focus should be on the highest-prevalence areas and high-risk populations. No nationwide studies were identified in this meta-analysis from 2018 to 2022, there is a need for more systematic estimates of nationwide HBV prevalence.

### Supplementary Information

**Additional file 1. **Search Term

**Additional file 2: Table S1.** Cross-Sectional/Prevalence Study Quality Assessment Forms

**Additional file 3: Table S2. **Characteristics of age groups of the studies included in the meta-analysis

**Additional file 4: Table S3.** Quality assessment of eligible studies

## Data Availability

The datasets used and analyzed during the current study are available from the corresponding author on reasonable request.

## Abbreviations

HBV: Hepatitis B virus
HCV: Hepatitis C Virus
HBsAg: Hepatitis B surface antigen
PRISMA: Preferred Reporting Items for Systematic Reviews and Meta-Analyses
CNKI: China National Knowledge Infrastructure
CBM: Chinese Biomedical Database
HIV: Human Immunodeficiency Virus
AHRQ: Agency for Healthcare Research and Quality
CHB: Chronic Hepatitis B
HBeAg: Hepatitis B e-Antigen
ELISA: Enzyme-Linked Immunosorbent Assay
CMIA: CLIA-Chemiluminescence Immunoassay

## References

[1] Whiteford HA, Degenhardt L, Rehm J, Baxter AJ, Ferrari AJ, Erskine HE (2013). Global burden of disease attributable to mental and substance use disorders: findings from the global burden of disease study 2010. Lancet..

[2] Trépo C, Chan HL, Lok A (2014). Hepatitis B virus infection. Lancet..

[3] Nguyen MH, Wong G, Gane E, Kao J-H, Dusheiko G (2020). Hepatitis B virus: advances in prevention, diagnosis, and therapy. Clin Microbiol Rev..

[4] Schweitzer A, Horn J, Mikolajczyk RT, Krause G, Ott JJ (2015). Estimations of worldwide prevalence of chronic hepatitis B virus infection: a systematic review of data published between 1965 and 2013. Lancet..

[5] André F (2000). Hepatitis B epidemiology in Asia, the middle east and Africa. Vaccine..

[6] Shepard CW, Simard EP, Finelli L, Fiore AE, Bell BP (2006). Hepatitis B virus infection: epidemiology and vaccination. Epidemiol Rev..

[7] Cui F, Shen L, Li L, Wang H, Wang F, Bi S (2017). Prevention of chronic hepatitis B after 3 decades of escalating vaccination policy, China. Emerg Infect Dis..

[8] Liang X, Bi S, Yang W, Wang L, Cui G, Cui F (2009). Epidemiological serosurvey of hepatitis B in China—declining HBV prevalence due to hepatitis B vaccination. Vaccine..

[9] Wang H, Men P, Xiao Y, Gao P, Lv M, Yuan Q (2019). Hepatitis B infection in the general population of China: a systematic review and meta-analysis. BMC Infect Dis..

[10] Liu J, Wang X, Wang Q, Qiao Y, Jin X, Li Z (2021). Hepatitis B virus infection among 90 million pregnant women in 2853 Chinese counties, 2015-2020: a national observational study. Lancet Region Health-Western Pacif..

[11] Sun Q, Lao TT, Du M, Xie M, Sun Y, Bai B (2021). Chronic maternal hepatitis B virus infection and pregnancy outcome-a single center study in Kunming. China BMC Infect Dis..

[12] Moher D, Liberati A, Tetzlaff J, Altman DG, PRISMA Group* (2009). Preferred reporting items for systematic reviews and meta-analyses: the PRISMA statement. Ann Intern Med..

[13] Rostom A, Dubé C, Cranney A, Saloojee N, Sy R, Garritty C (2004). Celiac disease.

[14] R Core Team. R: A language and environment for statistical computing. Vienna: R Foundation for Statistical Computing; 2023. https://www.R-project.org/.

[15] National Bureau of Statistics of China: National Bureau of Statistics of China register. 2021. https://www.stats.gov.cn/zt_18555/zthd/sjtjr/d12kfr/tjzsqzs/202302/t20230216_1908940.html. Accessed 15 Feb 2023.

[16] National Bureau of Statistics of China: National Bureau of Statistics of China register. 2023. https://www.stats.gov.cn/hd/lyzx/zxgk/202401/t20240104_1946235.html. Accessed 15 Feb 2023.

[17] Luo Z, Li L, Ruan B (2012). Impact of the implementation of a vaccination strategy on hepatitis B virus infections in China over a 20-year period. Int J Infect Dis..

[18] Lin L, Xu C (2020). Arcsine-based transformations for meta-analysis of proportions: pros, cons, and alternatives. Health Sci Rep..

[19] Page MJ, Sterne JA, Higgins JP, Egger M (2021). Investigating and dealing with publication bias and other reporting biases in meta-analyses of health research: A review. Res Synth Methods..

[20] He Z, Xu R, You F, Zhou Z (2019). Epidemic status of hepatitis B and its influencing factors in people aged 1-59 years in Dali prefecture in 2018. Academ J Chin Pla Med School..

[21] Sun C (2019). Investigation and research on the carrier status of hepatitis B surface antigen among children aged 2 to 6 in a certain urban area and township. J Bethune Med Sci..

[22] He ZYF (2020). Seroepidemiological survey of hepatitis B among rural women in child-bearing age in Dali prefecture. J Hebei Med Univer..

[23] Deng Q, Zhong G, Liu W, Wei J, Yang R, Du J (2020). Sero-prevalence of hepatitis B among people aged 1-59 years in Guangxi Zhuang autonomous region, 2018. Chin J Vacc Immun..

[24] Li G (2020). Epidemiological status of viral hepatitis and related influencing factors. Chin J Public Health Eng..

[25] Shi W, Zhou Y, Yuan C, Yan R, Tang X, He H (2020). Seroepidemiological survey of hepatitis B in healthy population in Zhejiang, 2018. Dis Surveill..

[26] Li J, Tang T, Kang W, Xu L, Yu W, Li K (2021). Investigation on the status of hepatitis B virus infection and the vaccination situation among primary school students in Yunnan province. Int J Epidemiol Infect Dis..

[27] Li J, Xu L, Kang W, Yu W, Li K, Zhang J (2021). Seroprevalence for hepatitis B infection among children aged 8 months to 14 years in Yunnan province, 2019. Chin J Vacc Immun..

[28] Wang Q, Chen L, Liao J (2021). Serum epidemiological analysis of viral hepatitis B among people aged 1–69 years in Dazu district of Chongqing city. Bullet disease control prevent (china)..

[29] Xu N, Zhou X, Hu X, Zheng L, Fan C, Wen X (2021). Hepatitis B vaccine coverage and hepatitis B seroprevalence among children entering kindergarten in Xihu district, Hangzhou city in 2019–2020. Chin J Vacc Immun..

[30] Yang Q, Huang Y, Wang W, Zhang C, Xu J, Zhang Z (2021). Comparative analysis on seroprevalence of hepatitis B in Guangzhou in 2008 and 2018. Chin J Epidemiol..

[31] Yang X, Zhou Y, Zhang H, Zhang S, Huang L, Lin H (2021). Investigation and analysis on sero-epidemiology of hepatitis B in Fujian, China, 2019-2020. Strait J Prev Med..

[32] Zhang S, Li F, Li X, Shan L, Wang H, Ma L (2021). A hepatitis B seroprevalence survey in a 0-79-year-old healthy population of Zhengzhou city in 2020. Chin J Vacc Immun..

[33] Jiang M, Zhu B, Yao Q, Lou H, Zhang X (2021). Anti-HBs levels in children under the age of two years born to HBV carrier mothers after immunoprophylaxis: a multicenter cross-sectional study. BMC Pediatr..

[34] Ci P, Chen J, Yang Q, Tathy S, Garma L, Zheng Y (2022). Seroepidemiological survey of hepatitis B among children in Milin county of Tibet 2020. J Prev Med Inf..

[35] Deng X, Gao J, Hu Y, Lu P, Guo H (2022). Analysis of combination modes of HBV serological markers and HBV viral loads among population aged 1-69 years in Jiangsu Province. Jiangsu. J Prev Med..

[36] Guo Z, A K Hao Z, Zhao S, Wu J, RY D (2022). Hepatitis B seroprevalences among a 1-69-year-old population of Qinghai province in 2020. Chin J Vacc Immun..

[37] Huang Q, Liang Y, Huang X, Zheng T, Lu X, Pan J (2022). Seroprevalence of hepatitis B among a 1-69-year-old population in Nanjing city in 2020. Chin J Vacc Immun..

[38] Li W, Wang R, Li F, Chen X (2022). Analysis of seroepidemiological survey of hepatitis B among people aged 0-79 years in Zhengzhou from 2019 to 2021. J Méd Forum..

[39] Liang Y (2022). Analysis of HBV positive rate and hepatitis B surface antibody detection results in healthy population in Jiaozuo City. South China J Prev Med..

[40] Liu L, Qi Q, Li Y, Liu J, Tong W, Shen L (2022). Hepatitis B surface antigen seroprevalence and its influencing factors among 1–14 - year-old children in Sichuan province 2020. Chin J Vacc Immun..

[41] Wu F, He W, Guo S, Zhao H, Zheng M, Tan X (2022). Investigation on prevalence of HBsAg and HBsAb in population aged 1 to 69 years in Jiangxi Province in 2020. Mod Prev Med..

[42] Xie C (2022). Seroepidemiological investigation of hepatitis B and hepatitis C in Zaozhuang City. Chin J Public Health Eng..

[43] Yang J, Dai D, Yao Q, Liang Y, Zhang L, Shi C (2022). A hepatitis B sero-epidemiological survey of a 1-59-year-old dong nationality population in northern Guangxi Zhuang autonomous region in 2021. Chin J Vacc Immun..

[44] Zhang T, Liu C, Zheng L, Wang L, Cai K (2022). Seroprevalence of hepatitis B among a 1–69 -year-old population of Hubei province in 2020. Chin J Vacc Immun..

[45] Cui F, Woodring J, Chan P, Xu F (2018). Considerations of antiviral treatment to interrupt mother-to-child transmission of hepatitis B virus in China. Int J Epidemiol..

[46] Xu X, Wu C, Lou Z, Peng C, Jiang L, Wu T (2023). Changing incidence of hepatitis B and persistent infection risk in adults: a population-based follow-up study from 2011 in China. BMC Pub Health..

[47] He L, Pan X, Yang J, Ma Q, Jiang J, Wang W (2020). HIV risk behavior and HIV testing among rural and urban men who have sex with men in Zhejiang Province, China: A respondent-driven sampling study. PLoS One..

[48] Wang Y, Liu H, Zhao M, Feldman MW, Williams AB (2020). Sex with partners met online: risky sexual behavior among bachelors in rural China. Aids Care-Psychol Socio-Med Asp Aids-Hiv..

[49] Goyal A, Murray JM (2017). Roadmap to control HBV and HDV epidemics in China. J Theor Biol..

[50] Li M, Zu J, Shen M, Zhuang G, Chen S, Wang F (2021). Evaluating the independent influence of sexual transmission on HBV infection in China: a modeling study. BMC Pub Health..

[51] Gao X, Wang X (2020). The trends of migration in China, 1949–2019. Chin Popul Dev Stud..

[52] Liu J, Zhang S, Wang Q, Shen H, Zhang M, Zhang Y (2016). Seroepidemiology of hepatitis B virus infection in 2 million men aged 21–49 years in rural China: a population-based, cross-sectional study. Lancet Infect Dis..

[53] Hwang EW, Cheung R. Global epidemiology of hepatitis B virus (HBV) infection. North Am J Med Sci. 2011;4(1)

[54] World Health Organization. Guidelines for the prevention care and treatment of persons with chronic hepatitis B infection: Mar-15: World Health Organization. 2015. 26225396

[55] Wang Y, Zhou H, Zhang L, Zhong Q, Wang Q, Shen H (2017). Prevalence of chronic hepatitis B and status of HBV care among rural women who planned to conceive in China. Sci Rep..

[56] Ott JJ, Stevens GA, Wiersma ST (2012). The risk of perinatal hepatitis B virus transmission: hepatitis B e antigen (HBeAg) prevalence estimates for all world regions. BMC Infect Dis..

[57] Zheng J, Li Q, Wang J, Zhang G, Wangen KR (2017). Inequality in the hepatitis B awareness level in rural residents from 7 provinces in China. Human Vaccin Immunotherap..

[58] Zhu D, Guo N, Wang J, Nicholas S, Wang Z, Zhang G (2018). Socioeconomic inequality in hepatitis B vaccination of rural adults in China. Human Vaccines Immun..

[59] Wang X, Liu J, Wang Q, Qiao Y, Jin X, Li Z, et al. Economic-related inequalities in hepatitis B virus infection among 115.8 million pregnant women in China from 2013 to 2020. E Clin Med. 2022:49. 10.1016/j.eclinm.2022.101465. 10.1016/j.eclinm.2022.101465 PMC9124701 35747197

[60] Zhou Y-H, Wu C, Zhuang H (2009). Vaccination against hepatitis B: the Chinese experience. Chin Med J..

[61] Cui F, Purha T, Hadler S, Liang X (2007). Analysis on new born hepatitis B immunization coverage and pregnant women hospital delivery rate in different regions. Chin J Vacc Immun..

[62] Tanaka M, Katayama F, Kato H, Tanaka H, Wang J, Qiao YL (2011). Hepatitis B and C virus infection and hepatocellular carcinoma in China: a review of epidemiology and control measures. J Epidemiol..

[63] Cui F, Liang X, Gong X, Chen Y, Wang F, Zheng H (2013). Preventing hepatitis B though universal vaccination: reduction of inequalities through the GAVI China project. Vaccine..

[64] The Central People's Government of the People's Republic of China Ministry of health: The Central People's Government of the People's Republic of China Ministry of health register. 2011. https://www.gov.cn/jrzg/2011-08/13/content_1925134.htm. Accessed 2 Feb 2023.

[65] Ma X. 1 billion yuan to achieve full coverage of blood station nucleic acid detection China’s blood safety supply ranks in the forefront of the world. 2017. https://www.yicai.com/news/5368782.html. Accessed 6 Feb 2023.

[66] Chen S, Mao W, Guo L, Zhang J, Tang S. Combating hepatitis B and C by 2030: achievements, gaps, and options for actions in China. BMJ Glob Health. 2020;5(6) 10.1136/bmjgh-2020-002306. 10.1136/bmjgh-2020-002306 PMC7328743 32605935

[67] Zhang SX, Dang RB, Zhang WD, Liang XF, Cui FQ. Analysis on economic efficacy regarding previous strategies and current recommendations for vaccination against hepatitis B virus in China. Chin J Epidemiol. 2008;1003–8. 19173882

[68] Liu J, Liang W, Jing W, Liu M (2019). Countdown to 2030: eliminating hepatitis B disease, China. Bull World Health Organ..

[69] Geretti AM, Austin H, Villa G, Smith C, Sabin C, Tsang R (2023). Hepatitis B virus infection in general practice across England: an analysis of the Royal College of general practitioners research and surveillance Centre real-world database. J Infect..

