# Supplementary material for: The prevalence of hepatitis B in Chinese general population from 2018 to 2022: a systematic review and meta-analysis
Source: BMC Infect Dis. 2024 Feb 16;24:211. doi: 10.1186/s12879-024-09103-8 (PMC10870619; doi:10.1186/s12879-024-09103-8)
Supplement: Supplementary file 1 — Additional file 1. Search Term [file 12879_2024_9103_MOESM1_ESM.pdf]

ADDITIONAL FILE 1

Search Term

In Embase:

1. HBsAg:ab,ti OR ‘hepatitis B’:ab,ti OR ‘Hepatitis B virus’:ab,ti OR HBV:ab,ti

2. prevalence:ab,ti OR prevalent:ab,ti OR epidemic:ab,ti OR epidemiology:ab,ti OR carrier:ab,ti OR ‘positive rate’:ab,ti OR ‘infection rate’:ab,ti

3. China:ab,ti OR Chinese:ab,ti

4. #1 AND #2 AND #3 AND [2018-2022]/py

In Web of Science:

1.(((((((TI=(HBsAg)) OR AB=(HBsAg)) OR TI=(hepatitis B)) OR AB=(hepatitis B)) OR TI=(Hepatitis B virus)) OR AB=(Hepatitis B virus)) OR TI=(HBV)) OR AB=(HBV)

2.(((((((((((((TI=(prevalence)) OR AB=(prevalence)) OR TI=(prevalent)) OR AB=(prevalent)) OR TI=(epidemic)) OR AB=(epidemic)) OR TI=(epidemiology)) OR AB=(epidemiology)) OR TI=(carrier)) OR AB=(carrier)) OR TI=(positive rate)) OR AB=(positive rate)) OR TI=(infection rate)) OR AB=(infection rate)

3.(((TI=(Chinese)) OR AB=(Chinese)) OR TI=(China)) OR AB=(China)

4.#3 AND #2 AND #1

In Cochrane Database of Systematic Reviews:

1. HBsAg.ti,ab

2. ‘hepatitis B’.ti,ab

3. ‘Hepatitis B virus’.ti,ab

4. HBV.ti,ab

5. ‘Hepatitis B Surface Antigens’.sh

6. ‘Hepatitis B’.ti,ab

7. 1 OR 2 OR 3 OR 4 OR 5 OR 6

8. prevalence.ti,ab

9. prevalent.ti,ab

10. epidemic.ti,ab

11. epidemiology.ti,ab

12. carrier.ti,ab

13. ‘positive rate’.ti,ab

14. ‘infection rate’.ti,ab

15. Prevalence.sh

16. Epidemiology.sh

17. 8 OR 9 OR 10 OR 11 OR 12 OR 13 OR 14 OR 15 OR 16

18. China.ti,ab

19. Chinese.ti,ab

20. 18 OR 19

21. 7 AND 17 AND 20

In Medline through EBSCOhost

1. HBsAg.ti,ab

2. ‘hepatitis B’.ti,ab

3. ‘Hepatitis B virus’.ti,ab

4. HBV.ti,ab

5. ‘Hepatitis B Antibodies’.sh

6. ‘Hepatitis B virus’.sh

7. ‘Hepatitis B’.sh

8. ‘Hepatitis B Antigens’.sh

9. ‘Hepatitis B Surface Antigens’.sh

10. 1 OR 2 OR 3 OR 4 OR 5 OR 6 OR 7 OR 8 OR 9

11. prevalence.ti,ab

12. prevalent.ti,ab

13. epidemic.ti,ab

14. epidemiology.ti,ab

15. carrier.ti,ab

16. ‘positive rate’.ti,ab

17. ‘infection rate’.ti,ab

18. Epidemiology.sh

19. Epidemics.sh

20. Prevalence.sh

21. 11 OR 12 OR 13 OR 14 OR 15 OR 16 OR 17 OR 18 OR 19 OR 20

22. Chinese.ti,ab

23. China.ti,ab

24. China.sh

25. 22 OR 23 OR 24

26. 10 AND 21 AND 2

In Chinese databases (WanFang data, CNKI, CBM), the following keywords were used in Chinese:

[‘hepatitis B’ OR ‘HBsAg’] AND [‘prevalence’ OR ‘carrier’] AND [‘Chinese’ OR ‘China’] AND [(‘2018’ [Date - Publication]: ‘2022’[Date - Publication])]
