# Supplementary material for: The prevalence of hepatitis B in Chinese general population from 2018 to 2022: a systematic review and meta-analysis
Source: BMC Infect Dis. 2024 Feb 16;24:211. doi: 10.1186/s12879-024-09103-8 (PMC10870619; doi:10.1186/s12879-024-09103-8)
Supplement: Supplementary file 4 — Additional file 4: Table S3. Quality assessment of eligible studies [file 12879_2024_9103_MOESM4_ESM.docx]

**ADDITIONAL FILE 4**

**Table S3 Quality assessment of eligible studies**

|  |  |  |  |  | **Item** |  |  |  |  |  |  |
| --- | --- | --- | --- | --- | --- | --- | --- | --- | --- | --- | --- |
| **First author** | **①** | **②** | **③** | **④** | **⑤** | **⑥** | **⑦** | **⑧** | **⑨** | **⑩** | **Score** |
| He Z [20] | Y | UC | Y | Y | UC | N | N | Y | N | Y | 5 |
| Sun CW [21] | Y | Y | Y | Y | UC | N | N | N | N | Y | 5 |
| He Z [22] | Y | Y | Y | Y | UC | N | N | N | N | Y | 5 |
| Deng QY [23] | Y | N | Y | Y | UC | N | N | N | N | Y | 4 |
| Li GD [24] | Y | Y | N | Y | UC | N | N | Y | N | Y | 5 |
| Shi W [25] | Y | Y | Y | Y | UC | N | N | N | N | Y | 5 |
| Li JR [26] | Y | N | Y | Y | UC | Y | N | Y | N | Y | 5 |
| Li JR [27] | Y | UC | Y | Y | UC | N | N | N | N | Y | 4 |
| Wang Q [28] | Y | UC | Y | Y | UC | N | N | Y | N | Y | 5 |
| Xu NN [29] | Y | N | Y | Y | UC | N | N | N | N | Y | 4 |
| Yang QY [30] | Y | Y | Y | Y | UC | N | N | N | N | Y | 5 |
| Yang XH [31] | Y | N | Y | Y | UC | N | N | N | N | Y | 4 |
| Zhang SY [32] | Y | N | Y | Y | UC | N | N | N | N | Y | 4 |
| M. Jiang [33] | Y | Y | Y | Y | UC | N | N | N | N | Y | 5 |
| Ci P [34] | Y | Y | Y | Y | UC | N | N | N | N | Y | 5 |
| Deng XY [35] | Y | N | Y | Y | UC | N | N | Y | N | Y | 5 |
| Guo ZZ [36] | Y | N | Y | Y | UC | N | N | N | N | Y | 4 |
| Huang QM [37] | Y | N | Y | Y | UC | N | N | N | N | Y | 4 |
| Li WQ [38] | Y | Y | Y | Y | UC | N | N | Y | N | Y | 6 |
| Liang Y [39] | Y | Y | Y | Y | UC | N | N | UC | N | Y | 5 |
| Liu LJ [40] | Y | UC | Y | Y | UC | N | Y | N | N | Y | 5 |
| Wu FY [41] | Y | UC | Y | Y | UC | N | N | N | N | Y | 4 |
| Xie CZ [42] | Y | N | Y | Y | UC | N | N | N | N | Y | 4 |
| Yang JX [43] | Y | UC | Y | Y | UC | N | N | N | N | Y | 4 |
| Zhang T [44] | Y | N | Y | Y | UC | Y | N | N | N | Y | 5 |

Y: Yes; N: No; UC: Unclear.
